# SlPPR138-mediated RNA editing of *rpoC1* is essential for chloroplast development in tomato

**DOI:** 10.1093/hr/uhaf194

**Published:** 2025-07-28

**Authors:** Yichen Liu, Chengwen Li, Xiuyang Si, Tao Zou, Ye Li, Changtian Pan, Gang Lu

**Affiliations:** Department of Horticulture, College of Agriculture and Biotechnology, Zhejiang University, Hangzhou 310058, China; Department of Horticulture, College of Agriculture and Biotechnology, Zhejiang University, Hangzhou 310058, China; Department of Horticulture, College of Agriculture and Biotechnology, Zhejiang University, Hangzhou 310058, China; Department of Horticulture, College of Agriculture and Biotechnology, Zhejiang University, Hangzhou 310058, China; Department of Agronomy, HeiLongJiang Agricultural Engineering Vocational College, Harbin 150088, China; Department of Horticulture, College of Agriculture and Biotechnology, Zhejiang University, Hangzhou 310058, China; Zhejiang Key Laboratory of Horticultural Crop Quality Improvement, Zhejiang University, Hangzhou 310058, China; Department of Horticulture, College of Agriculture and Biotechnology, Zhejiang University, Hangzhou 310058, China; Key Laboratory of Horticultural Plant Growth, Development and Quality Improvement, Ministry of Agricultural, Zhejiang University, Hangzhou 310058, China

## Abstract

Leaf color is a crucial determinant of photosynthetic efficiency and crop yield, but the molecular mechanisms regulating chloroplast development in tomato remain incompletely understood. Here, we identified a novel tomato mutant, *gret1*, that exhibits yellow cotyledons and young leaves that gradually turn green upon maturation. The *gret1* mutant displays significantly reduced chlorophyll content and defective chloroplast development at early leaf stages, accompanied by changes in expression of genes involved in photosynthesis and chloroplast biogenesis. Genetic analysis revealed that the *gret1* phenotype is controlled by a single recessive nuclear gene. Using map-based cloning, we identified *SlPPR138*, encoding a DYW-type pentatricopeptide repeat (PPR) protein, as the causal gene. A T-to-C point mutation in *SlPPR138* causes a Cys-to-Arg substitution, which disrupts its function. Both genetic complementation and CRISPR/Cas9 knockout experiments validated that the *gret1* phenotype is caused by the loss of SlPPR138. Mechanistically, we found that SlPPR138 mediates chloroplast RNA editing, particularly affecting the C-to-U editing efficiency of *rpoC1*, which encodes a core subunit of plastid-encoded RNA polymerase (PEP) complex. These findings demonstrate SlPPR138 is essential for early chloroplast development through RNA editing, providing new insights into the post-transcriptional regulation of photosynthesis in plants.

## Introduction

Leaves are pivotal for plant growth as the primary organs for photosynthesis—a complex process essential for energy conversion and environment adaptation. Chloroplasts, the specialized organelles where photosynthesis takes place, harness solar energy to drive anabolic reactions and export metabolic products [1, 2]. The formation and maintenance of functional chloroplasts requires precise spatiotemporal regulation at multiple levels, impacting plant growth, crop yield, and quality [3, 4]. Therefore, a deep deciphering of chloroplast biogenesis is critical for improving photosynthetic efficiency and crop productivity [5].

Chloroplast biogenesis is a complex and finely regulated process that begins with the differentiation of proplastids into mature chloroplasts. This transformation occurs through three coordinated phases: (i) plastid DNA synthesis and replication, (ii) establishment of the chloroplast genetic system, and (iii) assembly of photosynthetic apparatus [6]. Each of these stages is meticulously coordinated by two essential RNA polymerases, the nuclear-encoded RNA polymerase (NEP) and the plastid-encoded RNA polymerase (PEP). NEP, a single-subunit phage-type RNA polymerase, primarily drives the transcription of genes essential for early chloroplast biogenesis [7, 8]. Conversely, PEP, a multisubunit enzyme consisting of core subunits (*α*, *β*, *β′*, and *β″*) encoded by chloroplast *rpo* genes (*rpoA*, *rpoB*, *rpoC1*, and *rpoC2*), mainly transcribes genes associated with photosynthesis and plastid tRNAs [9–11]. Plastid-encoded genes are divided into three classes based on their corresponding RNA polymerases: Class I genes are transcribed solely by PEP (e.g. *psaA*, *psbA*, *rbcL*), Class II genes by both NEP and PEP (e.g. *atpB*, *atpI*, *ndhB*, *ndhF*, *clpP*, *ycf1*), and Class III genes exclusively by NEP (e.g. *rpoA*, *rpoB*, *rpoC1*, *rpoC2*) [12]. PEP activity is particularly vital for light-responsive gene expression within the chloroplast genome. The absence of PEP core subunits often leads to severe defects in chloroplast development, resulting in pigment deficiencies or seeding lethality [13].

Chloroplast formation has been extensively studied in model plants such as *Arabidopsis* and rice, utilizing various leaf color mutations to investigate the process. In rice, the *ygl19* mutant exhibits a yellow-green phenotype with reduced chlorophyll content and photosynthetic efficiency [14]. The *asl3* mutant presents a lethal albino phenotype during the seedling stage due to disrupted chloroplast development [15]. Albino phenotypes typically arise from reduced PEP or NEP activity during the early stages of chloroplast development. For instance, the rice *OsPAP3* gene is known to regulate chloroplast development by activating PEP-mediated gene expression in chloroplasts [16]. In *Arabidopsis*, *PD1* mutants exhibit albino phenotypes due to disrupted PEP-β accumulation [13]. Additionally, recent research has identified several key regulators of PEP activity that are located in the nucleus. These include members of the pentatricopeptide repeat (PPR) protein family, such as *BoYgl-2* [17], *OsGLK1* [18], and *AtECB2* [19], that play essential roles in the editing or splicing of *rpo* genes, thereby influencing the proper assembly and function of PEP core subunits. Although these studies have advanced our understanding of chloroplast development, the mechanisms underlying reversible chlorophyll deficiencies—particularly those linked to RNA editing—remain poorly understood.

Tomato (*Solanum lycopersicum* L.) is a widely produced vegetable and serves as an excellent model for studying chloroplast development and photosynthesis. The first documented tomato leaf color mutant, *gh*, reported by Mackinney *et al*., is characterized by unstable chlorophyll deficiency [20]. Subsequently, the naturally occurring yellow leaf mutant (*ym*) demonstrates a yellowing phenotype due to compromised chloroplast structure, diminished pigment content, and impaired reactive oxygen species (ROS) scavenging capabilities [21, 22]. Notable mutants, such as *lutescent1* (*l1*) and *lutescent2* (*l2*), have provided insights into chlorophyll metabolism and chloroplast function, exhibiting chlorophyll deficiencies characterized by reduced synthesis and accelerated degradation under conditions of high light stress and darkness [23]. Additionally, the recent discovery of the variegated-leaf mutant (*vg*) has shed light on the role of thylakoid formation proteins in regulating chlorophyll synthesis and photosynthesis [24], while the white virescent mutant (*wv*) presents white virescent apical buds and immature leaves, sensitive to temperature and light [25]. Recent discoveries, such as the mottled leaf mutant found by Dechkrong *et al*. with mottled leaves from the M_2_ population of EMS-mutagenized seeds, continue to contribute to our comprehension of chlorophyll production and chloroplast functionality [26]. Although these studies collectively deepen our understanding of chlorophyll biosynthesis and chloroplast regulation, the molecular mechanisms underlying these phenotypes remain largely elusive.

Here, we characterized a novel tomato mutant, *gret1*, that displays a developmentally reversible chlorophyll-deficient phenotype. Initially, the cotyledons and newly emerged leaves of the *gret1* mutant displayed a yellowish phenotype that gradually turned green as the leaves mature, suggesting the presence of a previously uncharacterized regulatory checkpoint in chloroplast development. Through map-based cloning, we pinpointed the gene *SlPPR138*, responsible for encoding a DYW-type PPR protein, as the key player behind the mutant phenotype. A single T-to-C transition in *SlPPR138* results in a cysteine to arginine (Cys-to-Arg) substitution that impairs its function. Subsequent real-time polymerase chain reaction (RT-PCR) and Sanger sequencing analyses revealed that RNA editing of the chloroplast gene *rpoC1* is partially inhibited in the *gret1* mutant and *SlPPR138* knockout lines. These findings shed light on the vital role for *SlPPR138* in regulating RNA editing and facilitating chloroplast formation in tomato plants.

## Results

### Identification and phenotypic characterization of the *gret1* mutant

**Figure 1:**
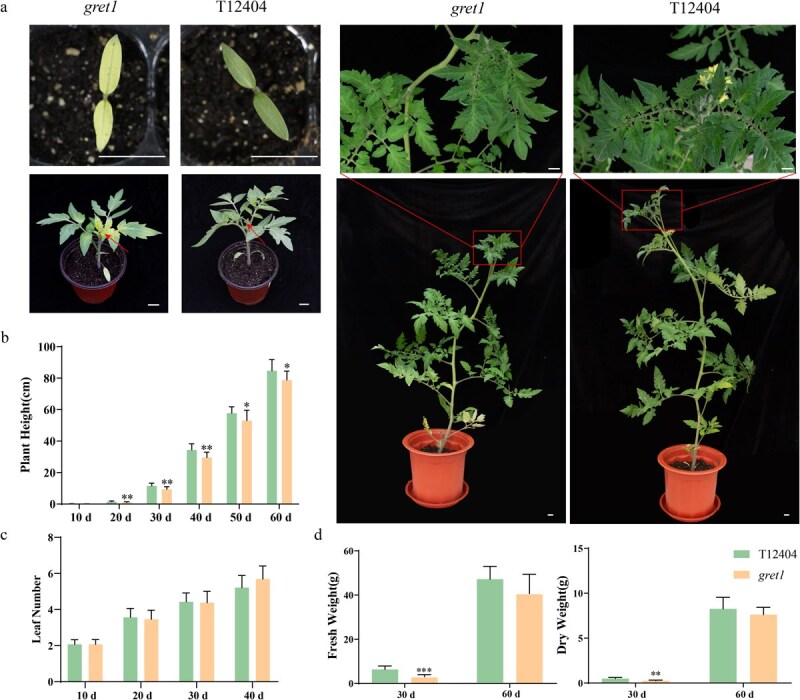
The contrasting phenotypes of WT (T12404) and *gret1* mutant tomato plants. (a) Whole-plant morphology of *gret1* and WT at different growth stages. Scale bars: 5 cm. (b) Plant height of WT and *gret1* mutant at different growth stages. (c) Leaf number of WT and *gret1* mutant. (d) Fresh and dry weight of WT and *gret1* mutant at 30 DAG and 60 DAG. Error bars indicate ±SD (*n* = 5). The following is an indication of significance: ^*^P < 0.05; ^**^P < 0.01, ^***^P < 0.001 (Student’s *t*-test)

We isolated a novel tomato mutant, *gret1*, from the breeding line ‘T12404’, exhibiting a unique green-revertible etiolation phenotype. In *gret1* plants, the cotyledons show severe etiolation, whereas the emerging leaves initially appear yellowish but gradually turn green as they mature (Fig. 1a). By the fourth or fifth leaf, a light yellow-green hue becomes noticeable, eventually stabilizing to green in later leaves (Fig. 1a), suggesting stage-specific chloroplast dysfunction. This *gret1* mutant is the first documented case of a green-revertible etiolation mutant in tomatoes, having been self-pollinated for more than five generations prior to further analysis (Fig. 1a).

Agronomic assessments indicated that *gret1* plants exhibited a distinct dwarf phenotype in the early stages of growth, showing a noticeable decrease in plant height when compared to their wild-type (WT) counterparts (Fig. 1b). Despite this stunted growth, the number of leaves on *gret1* plants remained consistent with those seen in WT plants (Fig. 1c). By 30 days after germination (DAG), both the aboveground biomass’ dry and fresh weights of the aboveground biomass in *gret1* plants were significantly reduced, by ~50% and 48%, respectively (Fig. 1d). Once leaf color normalized, *gret1* plants began to exhibit growth patterns that closely resembled those of the WT, including typical flowering and fruit-setting behaviors by 60 DAG (Fig. S1). Additionally, the final traits of fruits and seeds, such as fruit weight, transverse and longitudinal diameters, locule number, and soluble solid content, were comparable between *gret1* and WT plants (Fig. S1).

### The *gret1* mutant affects leaf photosynthesis and chloroplast development

**Figure 2:**
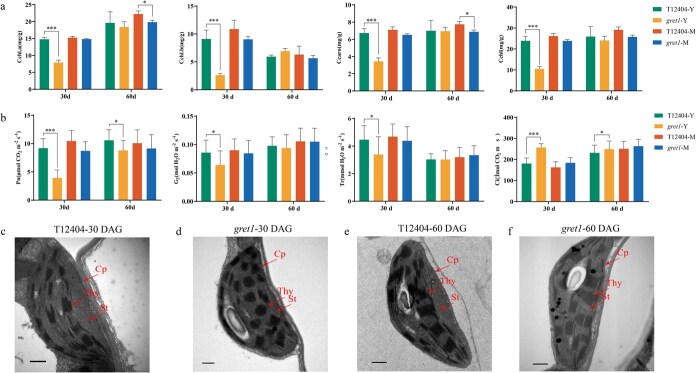
Measurement of leaf photosynthetic parameters and chloroplast ultrastructure in WT and *gret1* plants at different stages. (a) Leaf photosynthetic pigment content in WT and *gret1* plants at 30 and 60 DAG. Measurements include total chlorophyll (Chl), chlorophyll a (Chl a), chlorophyll b (Chl b), and carotenoids (Car). Y, young leaves. M, mature leaves. (b) Photosynthetic parameters in WT plants at various growth stages. (c) Chloroplast ultrastructure in *gret1* plants at 30 DAG. (d) Chloroplast ultrastructure in WT and *gret1* plants at 30 DAG. Note the differences in grana and thylakoid organization. Cp, Chloroplast; St, Stroma; Thy, Thylakoid. Scale bars: 0.5 μm. (e) Chloroplast ultrastructure in WT plants at 60 DAG. (f) Chloroplast ultrastructure in *gret1* plants at 60 DAG. Scale bars: 0.5 μm. Error bars indicate ±SD (*n* = 5). Statistical significance is indicated as follows: ^*^P < 0.05; ^**^P < 0.01, ^***^P < 0.001 (Student’s *t*-test).

To further investigate the *gret1* mutant, we delved into the impact on chlorophyll levels and photosynthetic function in both young and mature leaves compared to WT plants. At 30 DAG, the *gret1* young leaves retained only 44% of WT chlorophyll (Chl) levels (Chl a: 53%; Chl b: 29%) (Fig. 2a). Conversely, in fully developed leaves, the photosynthetic pigment content in *gret1* was akin to that of WT plants, indicating that the leaf-color phenotype in *gret1* is due to reduced photosynthetic pigments during early growth stages. We also assessed photosynthetic activity in the *gret1* mutant, finding that all measured photosynthetic parameters were significantly reduced, with net photosynthetic rate (Pn), stomatal conductance (Gs), and transpiration rate (Tr) decreasing by 57%, 24%, and 24%, respectively (Fig. 2b). Meanwhile, the intercellular CO_2_ concentration (Ci) saw a 30% increase, signifying a decline in photosynthetic efficacy during the early growth stages of the *gret1* mutant. (Fig. 2b).

Chloroplast ultrastructure was examined using transmission electron microscopy (TEM) at both early (30 DAG) and late (60 DAG) growth stages. At 30 DAG, chloroplasts in *gret1* leaves exhibited a morphology largely akin to that of WT, although with noticeable differences in thylakoid structure. The WT mesophyll cells had a highly differentiated inner membrane system with tightly packed granules and well-defined stromal thylakoids in a rectangular and organized manner. Conversely, *gret1* chloroplasts in immature yellow leaves displayed disorganized membrane structures; the grana lamellae within the stroma were irregularly stacked, and the thylakoids appeared rounded with a blurred structure (Fig. 2c and d). By 60 DAG, however, chloroplasts in *gret1* leaves showed well-organized thylakoids and stacked grana, resembling the WT (Fig. 2e and f). Together, these results demonstrate that the *gret1* mutation disrupts chloroplast development, impairing photosynthetic capacity in young leaves.

### Map-based cloning identifies *SlPP138* as the causal gene

**Figure 3:**
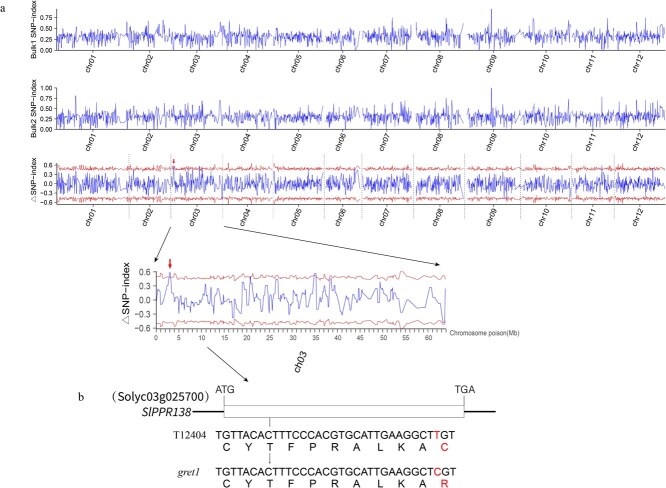
Map-based cloning of the *gret1* gene in tomato. (a) Allele frequency differences between the WT-like bulk and the yellowish-leaf bulk from the F_2_ population. The X-axis represents the 12 tomato chromosomes, and the Y-axis displays the difference in allele frequencies (mean SNP-index values) between the two bulks. The red line indicates the 99% confidence interval of the mean simulated SNP-index for mutant allele frequencies, with red arrows highlighting nonsynonymous mutations. (b) Gene structure of *SlPPR138*. Black lines represent introns and untranslated regions, while white boxes denote exons. The T-to-C substitution in the exon of *SlPPR138* results in an amino acid change from cysteine (Cys, C) in the WT to arginine (Arg, R) in the *gret1* mutant.

To determine the genetic foundation of the *gret1* mutant, we conducted a genetic analysis by crossing the *gret1* mutant with the WT line T12404. All F_1_ progeny exhibited the typical green phenotype, confirming that the *gret1* mutation is recessive. Analysis of the F_2_ population derived from these F_1_ individuals yielded 378 plants with normal green leaves and 105 plants displaying a yellowish phenotype in the cotyledons and early leaves. A genetic segregation of 3:1 (χ^2^ = 2.57, *P* < 0.05) indicates that a single recessive nuclear gene is responsible for the yellowish leaf phenotype (Table S2). Mapping of the *gret1* locus involved an F_2_ population from a cross between the *gret1* mutant and the WT. Two bulked DNA pools, composed of individuals with green and yellowish leaves, respectively, were sequenced. The sequence reads were aligned to the tomato reference genome, and differences in allele frequencies between the two pools were assessed. Single nucleotide polymorphisms (SNPs) were identified across the 12 tomato chromosomes, culminating in a Manhattan plot (Fig. 3a). Four SNPs within 99% confidence intervals were identified in regions: SL4.0ch03_3100000 to SL4.0ch03_3200000, SL4.0ch03_21400000 to SL4.0ch03_21500000, and SL4.0ch03_34900000 to SL4.0ch03_35800000 (Table S3). Among these, only the SNP between SL4.0ch03_3100000 and SL4.0ch03_3200000 led to a nonsynonymous amino acid change, while the others were situated in intergenic regions (Fig. 3).

Sequence comparison identified a causal T-to-C missense mutation at SL4.0ch03_3101674 in *gret1*, resulting in a Cys-to-Arg substitution (Fig. 3b). Genetic analysis of the F_2_ population confirmed monogenic recessive inheritance, mapping the leaf-color phenotype to Solyc03g025700. This gene encodes a PPR protein, which we designated *SlPPR138*, the 138th PPR gene in tomato according to Ding *et al*. (2014) [27].

### Complementation analysis and functional verification of *SlPPR138*

**Figure 4:**
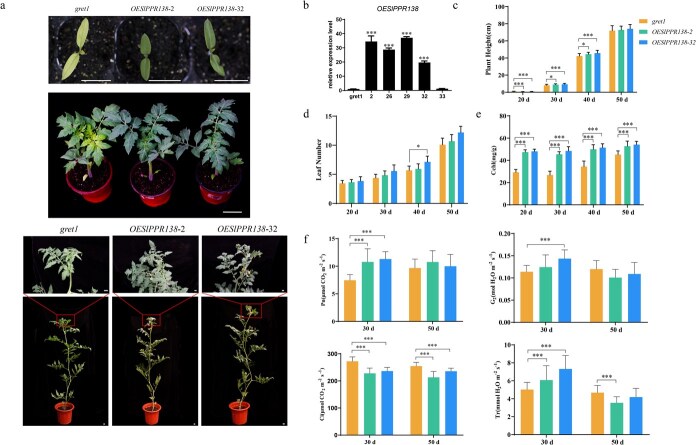
Complementation of the *gret1* mutant phenotype with the *SlPPR138* gene. (a) Whole-plant morphology of *SlPPR138* overexpression lines and the *gret1* mutant at various developmental stages. Scale bars: 5 cm. (b) Expression levels of *SlPPR138* in independent overexpression lines compared to the *gret1* mutant control. The *SlUbi3* gene was used as the internal control. Data are presented as means ± SD (*n* = 5). (c) Plant height of *gret1* mutant and *SlPPR138* overexpression lines from 20 to 50 DAG. (d) Leaf number of *gret1* mutant and *SlPPR138* overexpression lines from 20 to 50 DAG. (e) Photosynthetic pigment content in *gret1* mutant and *SlPPR138* overexpression lines at different developmental stages. (f) Photosynthetic parameters of mature leaves in *gret1* mutant and *SlPPR138* overexpression lines at 30 and 50 DAG. Data are presented as mean ± SD of five biological replicates. Statistically significant differences are shown by asterisks above the bars (Student’s *t*-test; ^*^P < 0.05, ^**^P < 0.01, ^***^P < 0.001).

In order to determine the role of the *SlPPR138* in causing the chlorophyll-deficient characteristics seen in the *gret1* mutant, we conducted a complementation experiment. We engineered an overexpression plasmid containing the full-length WT *SlPPR138* coding sequence under the control of the 35S CaMV promoter and introduced it into the *gret1* mutant. Twelve independent PCR-positive lines were produced. In comparison to the *gret1* mutant, these complemented lines displayed normal green leaf coloration (Fig. 4a). The expression levels of *SlPPR138* were notably elevated in the *OESlPPR138–2* and *OESlPPR138–32* lines (Fig. 4b). Furthermore, both chlorophyll content and photosynthetic parameters were substantially enhanced in these overexpression lines (Fig. 4c–f). Conversely, the *gret1* plants maintained their chlorophyll-deficient traits, highlighting the effectiveness of the normal *SlPPR138* gene in correcting the etiolation phenotype.

**Figure 5:**
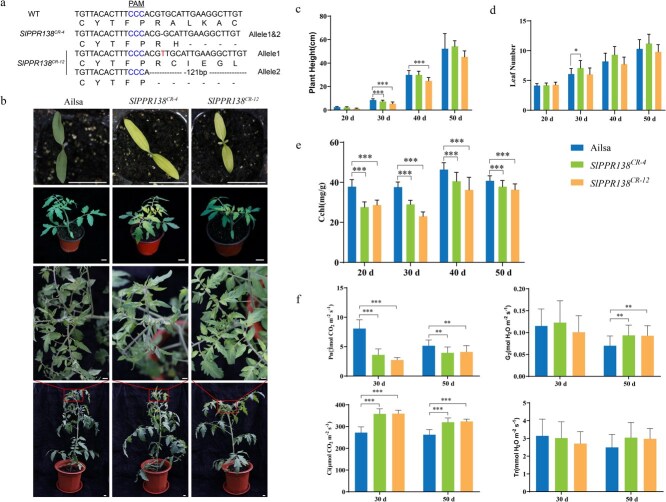
Phenotypes of *SlPPR138*-knockout lines compared to WT plants. (a) Identification of mutation types in *SlPPR138*-knockout lines within the T_1_ generation. (b) Whole-plant morphology of *SlPPR138*-knockout lines and the WT (Ailsa Craig, AC) at various developmental stages. Scale bars: 5 cm. (c) Plant height measured from 20 to 50 DAG. (d) Leaf number recorded from 20 to 50 DAG. (e) Photosynthetic pigment content assessed from 20 to 50 DAG. (f) Photosynthetic parameters of young leaves in WT plants and *SlPPR138*-knockout lines at 30 and 50 DAG, respectively. Data are presented as mean ± SD of five biological replicates. Statistically significant differences are shown by asterisks above the bars (Student’s *t*-test; ^*^P < 0.05, ^**^P < 0.01, ^***^P < 0.001).

To further understand the role of SlPPR138 in the development of leaves, we employed CRISPR/Cas9 technology to create *SlPPR138*-knockout lines in WT plants. We generated forty T_0_ transgenic plants, and genotypic analysis confirmed successful genetic modifications. The *SlPPR138^CR-4^* line exhibited a distinct homozygous 1-bp deletion in exon 1, leading to a frameshift mutation and a premature stop codon. The *SlPPR138^CR-12^* line featured a 1-bp insertion coupled with a 121-bp deletion, both leading to frameshifts and premature termination codons (Fig. 5a). The *SlPPR138*-knockout mutants initially displayed yellowing in the leaves before the 8th true leaf, which then transitioned to normal green coloration in later leaves (Fig. 5b). Plant height was reduced during the early growth stages (Fig. 5c), although the number of leaves remained unchanged (Fig. 5d). By 50 DAG, around the 10th leaf stage, the *SlPPR138*-knockout plants showed normal growth patterns akin to those of WT plants (Fig. 5b and c). The flowering and fruiting characteristics of the *SlPPR138*-knockout plants were also comparable to WT (Fig. S2).

Consequently, the absence of *SlPPR138* in plants resulted in a noteworthy decrease in total chlorophyll content in the new leaves, showing an 18% reduction compared to WT plants (Fig. 5e). Furthermore, the net photosynthetic rate decreased by 23%, while the transpiration rate increased by 25% compared to the WT (Fig. 5f). This emphasizes the significance of SlPPR138 in facilitating optimal chlorophyll synthesis and chloroplast growth in young tomato plants.

### *SlPPR138* encodes a DYW-type PPR protein found in chloroplasts

**Figure 6:**
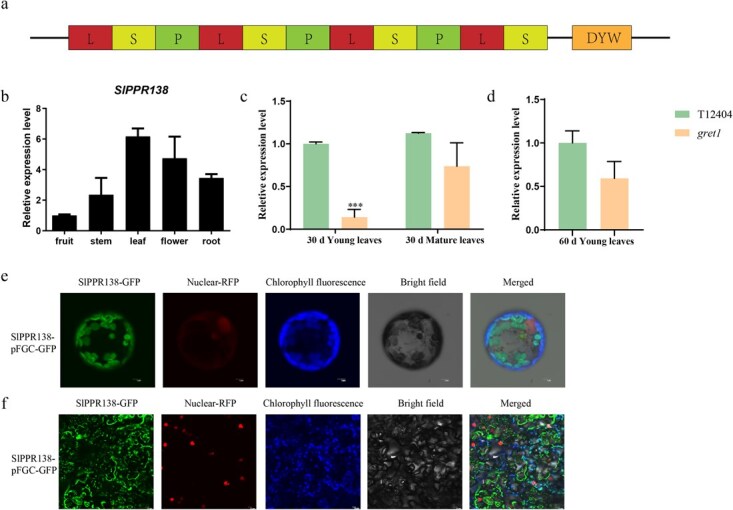
Expression profiles and subcellular localization analysis of *SlPPR138*. (a) Structure model of the SlPPR138 protein, which comprises 11 PPR motifs (P, L, S) and one additional structural domain (DYW). (b) Relative expression levels of *SlPPR138* in various tissues of tomato cultivar ‘Ailsa Craig’, including the root, stem, leaf, flower, and green mature fruit. *SlUbi3* was used as the internal control. Error bars represent the mean ± SD of three biological replicates. (c) Relative expression levels of *SlPPR138* in young and mature leaves of WT (T12404) and *gret1* mutant at 30 DAG. (d) Relative expression levels of *SlPPR138* in young leaves of WT (T12404) and *gret1* mutant in 60 DAG. (e) Subcellular location of the SlPPR138-GFP fusion protein in leaf epidermal cells of tomato protoplasts. GFP, green fluorescent protein; RFP: red fluorescent protein of nucleic marker. Chlorophyll, chlorophyll autofluorescence. Merge, merge images of GFP, RFP and chlorophyll autofluorescence. Scale Bars: 5 μm. (f) Subcellular location of the SlPPR138-GFP fusion protein in the leaf epidermal cells of *N. benthamiana*. Scale Bars: 5 μm.

*SlPPR138* encodes a 353-amino acid DYW-type PPR protein containing 11 characteristic PPR motifs (Fig. 6a). Quantitative RT-PCR (qRT-PCR) analysis *SlPPR138* was ubiquitously expressed across all examined tissues, with predominant transcript accumulation in leaves (Fig. 6b). Notably, *SlPPR138* expression was significant decreased reduced specifically in young leaves of the *gret1* mutant at 30 DAG (Fig. 6c and d). Subcellular localization confirmed that SlPPR138-GFP fluorescence exclusively colocalized with chloroplast fluorescence, in both tomato and *Nicotiana benthamiana* epidermal cells, verifying its chloroplast localization (Fig. 6e , f and Fig. S7).

### *SlPPR138* mutant exhibits decreased PEP activity during early leaf development

To determine if the expression of chlorophyll-related genes is impacted in the *gret1* mutant, we first analyzed the transcript levels of chloroplast development-related genes in both WT and *gret1* plants. Our findings indicated a significant decrease in the transcript levels of redox-related genes, including HEMF and CHLM, in the *gret1* mutant compared to the WT plants (Fig. S4a). This indicates a potential disruption in chlorophyll-related processes in the mutant plants.

**Figure 7:**
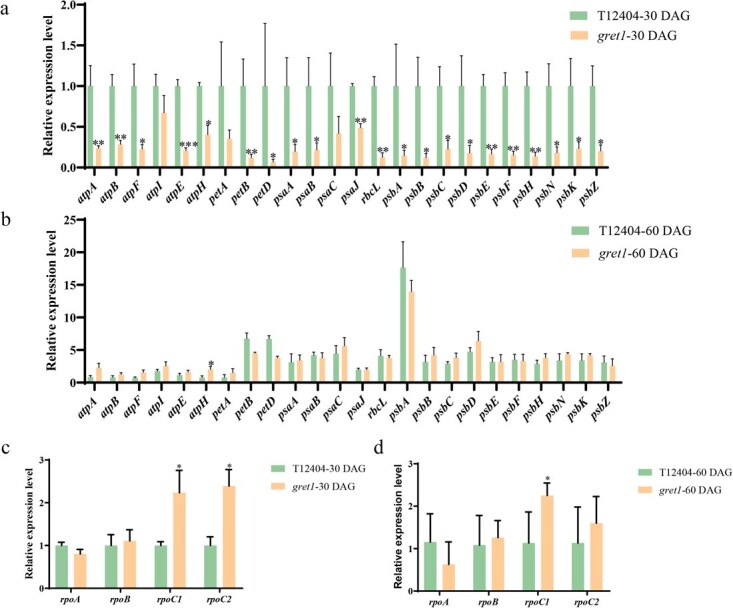
Expression of PEP-dependent and PEP core subunit genes in WT (T12404) and *gret1* mutant leaves. (a) PEP-dependent gene expression in WT and *gret1* leaves at 30 DAG. (b) PEP-dependent gene expression in WT (T12404) and *gret1* leaves at 60 DAG. (c) PEP-encoded gene expression in WT (T12404) and *gret1* leaves at 30 DAG. (d) PEP-encoded gene expression in WT (T12404) and *gret1* leaves at 60 DAG. *SlUbi3* was used as the reference gene. Data are presented as the mean ± SD of five biological replicates. Statistically significant differences are shown by asterisks above the bars (Student’s *t-*test: ^*^P < 0.05, ^**^P < 0.01).

At 30 DAG, the *gret1* mutant plants exhibited a significant decrease in the expression levels of Class I genes, primarily transcribed by PEP, in the newly developing leaves (Fig. 7a). Notably, the expression of *rbcL*, responsible for encoding the large subunit of Rubisco, also showed a marked reduction in the *gret1* mutant at 30 DAG (Fig. 7a). Rubisco activase (RCA), a nuclear-encoded soluble chloroplast enzyme [28, 29], also showed reduced levels in the *gret1* mutant at 30 DAG (Fig. 7a). However, by 60 DAG, the expression patterns of all PEP-dependent genes we investigated in the *gret1* mutant closely resembled those of the WT (Fig. 7b). These observations indicate that the *gret1* mutant exhibits defects in PEP activity during early leaf development.

Previous studies have shown the composition of PEP, which comprises four essential subunits produced by Class III genes, *rpoA*, *rpoB*, *rpoC1*, and *rpoC2*, that encode the *α*, *β*, *β’*, and *β”* subunits, respectively [9–11]. qRT-PCR analysis showed that the expression levels of *rpoC1* and *rpoC2* were substantially upregulated in *gret1* plants compared to WT plants at 30 DAG (Fig. 7c). However, by 60 DAG, only the *rpoC1* mRNA level remained significantly elevated in *gret1* plants, while *rpoA* expression was slightly reduced compared to the WT (Fig. 7d). These results suggest that the reduced PEP activity in the *gret1* mutant may be attributable to altered RNA levels.

### SlPPR138 is crucial for RNA editing of *rpoC1* during early leaf development

To elucidate SlPPR138’s role in chloroplast RNA metabolism, we first examined its potential involvement in the transcript splicing of chloroplast-related transcripts genes. Employing RT-PCR with specific primers (Table S1), we analyzed the splicing patterns of all 13 chloroplast-encoded genes between the *gret1* mutant and WT plants. Our findings revealed normal splicing patterns in *gret1* mutant and WT plants, except for the two showing minor, nonsignificant variations, unrelated to PEP function. Additionally, the expression level of the gene *rpoC1* was consistent between new and old leaves (Fig. S5a and b). These observations suggested SlPPR138 is not crucial for the splicing of these nuclear transcripts.

**Figure 8:**
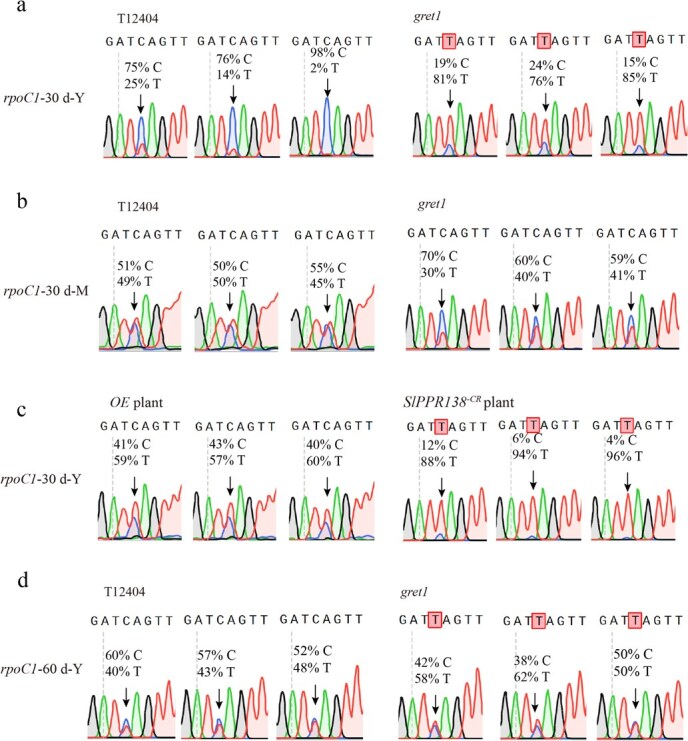
RNA editing efficiency of *rpoC1* in WT (T12404) and *gret1* mutant leaves. (a) Sequence chromatograms of plastid RNA editing sites of *rpoC1* in young leaves of WT (T12404) and *gret1* plants at 30 DAG. (b) Sequence chromatograms of plastid RNA editing sites of *rpoC1* in mature leaves of *gret1* plants at 30 DAG. (c) Sequence chromatograms of plastid RNA editing sites of *rpoC1* in young leaves of *SlPPR138*-overexpressing lines and *SlPPR138*-knockout lines at 30 DAG. (d) Sequence chromatograms of plastid RNA editing sites of *rpoC1* in young leaves of WT (T12404) and *gret1* plants at 60 DAG.

Given the notable changes in the expression of PEP-related genes, we next assessed RNA editing at eight known sites in four PEP core subunit genes [30]. Strikingly, the *gret1* exhibited ~60% reduction in *rpoC1* editing efficiency (T-to-C conversion) in the newly emerging leaves of *gret1* mutants at 30 DAG, whereas other PEP subunit genes remained unaffected (Fig. 8a, Fig. S6).

Conversely, the editing efficiency significantly increased to ~50% in mature leaves of the *gret1* mutant at 30 DAG, which is similar with WT levels (Fig. 8b). As anticipated, RNA editing efficiency of *rpoC1* in *SlPPR138*-knockout leaves was also decreased, indicating SlPPR138 knockout phenocopied the editing defect. On the other hand, the complemented plants (*OESlPPR138–2*), restored normal editing on *rpoC1* compared to *gret1* young leaves (Fig. 8c). At 60 DAG, the editing efficiency of *rpoC1* in young leaves in both *gret1* and WT plants was ~50% (Fig. 8d). These results demonstrate that SlPPR138 may ensure proper PEP-dependent transcription by specifically maintaining *rpoC1* RNA editing during early leaf development, and its dysfunction causes transient chloroplast defects in *gret1* mutants.

## Discussion

In this study, we characterized the green-revertible etiolation leaf mutant, *gret1*, which exhibits a distinct yellowish phenotype in cotyledons and newly emerging leaves during the early seedling stage. We observed a significant decrease in photosynthetic characteristics and a severe defect in chloroplast biogenesis in *gret1* mutant leaves (Fig. 2a–d). The *gret1* mutant exemplifies how leaf-color variants serve as genetic tools to dissect chloroplast development. Chloroplast damage typically results in altered leaf color and impaired photosynthesis, as seen in other mutants like maize’s *eal1* (etiolated/albino leaf 1), which also exhibits significant chloroplast defects [31]. Interestingly, while most characterized mutants (e.g. rice *ygl19* with necrotic spots or lethal albino [14]; *eal1* in maize [31]) exhibit irreversible defects, *gret1* leaf gradually reverts to green, and both photosynthetic activity and chloroplast development normalize (Fig. 2a–b, e–f). This developmental pattern of leaf color change is consistent with that observed in other mutants, such as the *ysa* mutant in *Arabidopsis*, which transitions from albinism before the three-leaf stage to green thereafter [32]. The *wsl9* mutant in rice exhibits white stripes during early leaf development but transitions to green from the four-leaf stage under field conditions [33].

Due to the close genetic relationship between naturally occurring mutants and their WT counterparts, crossing these mutants with WTs is an efficient method for gene isolation [34]. In this study, we used map-based cloning and allelic complementation unequivocally linked the *gret1* phenotype to a T-to-C missense mutation in *SlPPR138* (Fig. 3a). CRISPR-Cas9 knockout lines recapitulated the mutant phenotype (Fig. 5b–d), while complementation restored WT chlorophyll levels (Fig. 4c–f), firmly establishing the crucial function of SlPPR138 in chloroplast activity.

PPR proteins, initially identified in *Arabidopsis*, are characterized by a plant-specific structural motif that consists of 35 amino acids [35]. This family is divided into two main subfamilies: P and PLS (PPR-like) [36]. The PLS subfamily is further subdivided into three groups: E, E+, and DYW, based on variations in their C-terminal amino acid sequences. Research has shown that PPR proteins play a crucial role in chloroplast formation, with their absence leading to abnormal leaf color. For example, *OsPPR11*, a P-type PPR protein in rice, regulates the splicing of group II introns by interacting with the OsCAF2 protein, thereby influencing chloroplast development [37]. Another PPR protein, DUA1, interacts with sigma factor 1 (*OsSIG1*) to modulate chloroplast gene expression in response to environmental stimuli such as low temperature and low light [38]. In mitochondria and chloroplasts, these proteins bind to organelle transcripts and are crucial for RNA metabolism [39]. *SlPPR138* belongs to PLS DYW-type PPR protein located in the chloroplast with 11 PPR motifs. The PLS subfamily is particularly implicated in organellar RNA editing, with mutations often causing chloroplast defects [40]. For instance, OsPPR9 (DYW-type) edits multiple chloroplast transcripts, and its knockout is seedling-lethal in rice [41]. This functional divergence underscores SlPPR138’s specificity for RNA editing—a selectivity potentially dictated by its 11 PPR motifs’ recognition code [42].

In recent years, there has been a growing recognition of the importance of PPR proteins as key factors in RNA editing. Notably, Lan *et al*. shed light on the significant role played by the P-type PPR protein YLWS in rice [37]. This particular protein is essential for the early development of chloroplasts and has a significant impact on the RNA splicing and editing processes. Mutants exhibited temperature-sensitive, white-striped leaves and impaired chloroplast structures. Similarly, Chen *et al*. found that deleting the DYW-type PPR protein OsPPR9 in rice drastically reduced chloroplast RNA editing efficiency [41]. This led to defective chloroplast growth and photosynthesis, resulting in yellowish leaves and a lethal phenotype at the seedling stage. In *Arabidopsis*, the *OTP70* mutation causes an albino-lethal phenotype, likely due to splicing defects in the *rpoC1* gene [43]. In our study, we explored the role of *SlPPR138* in editing the chloroplast *rpoC1* gene, which is involved in chlorophyll synthesis. The *gret1* mutant had significantly lower *rpoC1* editing efficiency compared to the WT (Fig. 8), highlighting the importance of SlPPR138 in this process. The tomato *gret1* mutant primarily displayed yellowing in the cotyledons and young leaves, with symptoms lessening as the plant matured. Additionally, while *rpoC1* editing was significantly impaired in the *gret1* mutant, its splicing was less affected. Unlike constitutive lethal mutants (e.g. rice *OsPPR9*), SlPPR138’s impact is temporally constrained, suggesting editing redundancy emerges in the mature leaves.

In the *gret1* mutant, we observed significant defects in thylakoid development, particularly in the underdevelopment of grana lamellae and stroma lamellae (Fig. 2). This defect led to decreased photosynthesis-related parameters in early leaves and likely impacted chlorophyll synthesis. For instance, the *Arabidopsis* albino embryo and seedling mutant (*aes*), displays a breakdown of the chloroplast membrane system, decreased pigment content, and diminished photosynthetic activity due to reduced transcription levels of PEP-dependent chloroplast genes [44]. Similarly, the cucumber *alc* mutant displays milky green cotyledons under low light but perishes under normal light conditions, demonstrating how defects in chlorophyll metabolism genes can disrupt chlorophyll development and the photosynthetic system [45]. In the *gret1* mutant, PEP-dependent genes such as *psaA*, *psbA*, and *rbcL* showed reduced expression in newly emerging leaves at 30 DAG (Fig. 7a and c), indicating defective PEP activity in these early stages. By 60 DAG, the expression levels of these genes increased to approach WT levels, and leaf color returned to green (Fig. 7b and d), suggesting a normalization of PEP activity in older leaves. These results showed that the thylakoid defect in young *gret1* leaves (Fig. 2c–d) directly stems from PEP dysfunction due to impaired *rpoC1* editing (Fig. 8a), whereas the *gret1*’s PEP activity recovers by maturity in old leaves (Fig. 7b), which coincides with partial restoration of editing efficiency (Fig. 8b). We propose that transcriptional compensation (via NEP or other paralogs) mitigates early defects, enabling phenotypic recovery—a phenomenon with potential applications in engineering stress-resilient crops.

**Figure 9:**
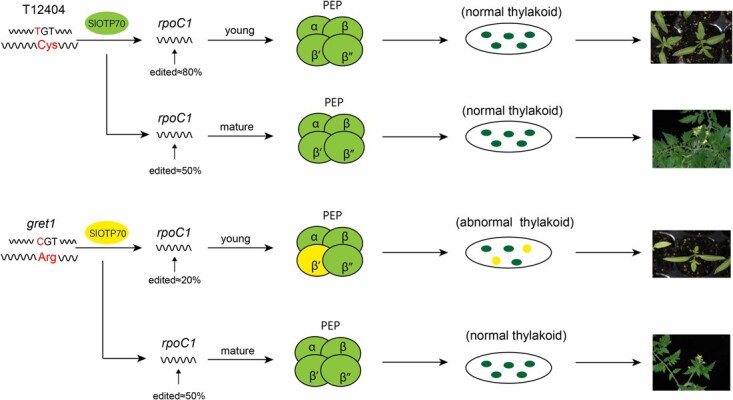
A schematic representation of the proposed mechanism for SlPPR138 in tomato chloroplast development.

Finally, we propose a mechanism for the role of SlPPR138 in tomato chloroplast development, as illustrated in Fig. 9. The *gret1* mutant exhibits a single nucleotide T-to-C substitution in the exon of SlPPR138, resulting in an amino acid shift from Cys (WT) to Arg (*gret1* mutant). This change does not alter the conformation of the SlPPR138 protein (Fig. S3). Nonetheless, we speculate that this amino acid alteration might compromise the protein’s ability to influence *rpoC1* editing. Consequently, this impairment affects the number of PEP β’ subunits, subsequently inhibiting the transcription of chloroplast-related genes and leading to the yellowish phenotype observed in early-stage leaves. There is a significant difference in editing efficiency in young leaves between *gret1* mutant and WT, but not in old leaves, indicating that the activity of PEP is restored as the plant matures, normalizing the leaf color. We hypothesize that functional redundancy with SlPPR138 homologs may compensate for its loss in mature tissues, as the plant matures, the editing efficiency remains at ~50% in the later stage of plant growth. Understanding the function of *SlPPR138* thus offers significant insights into the early processes of chlorophyll synthesis and chloroplast development in tomato leaves. This model informs strategies to engineer ‘recoverable’ chloroplast defects—enabling early-stage phenotyping without compromising yield, a paradigm valuable for high-throughput crop screening.

## Material and methods

### Plant material and growth conditions

The study employed the homozygous, high-generation tomato inbred line ‘T12404’ as the WT. The *gret1* mutant, a spontaneous yellow-green leaf variant, was isolated from an open-pollinated ‘T12404’ population and was self-pollinated for more than five generations before mapping. All tomato plants, including *N. benthamiana*, were cultivated under greenhouse conditions at Zhejiang University (Zhejiang, China), with a 16-h light period at 25°C followed by an 8-h dark period at 20°C to ensure optimal growth and development for the study.

### Determination of pigment content and photosynthetic parameters

Pigment content and photosynthetic parameters were assessed in the second true leaf (young leaf) and the second fully developed true leaf (old leaf) of both the *gret1* and ‘T12404’ lines at various growth stages. Photosynthetic parameters—including net photosynthetic rate (Pn), stomatal conductance (Gs), intercellular CO_2_ concentration (Ci), and transpiration rate (Tr)—were measured using an LI-6400 portable photosynthesis system (LI-COR, USA). Chlorophyll a, chlorophyll b, and carotenoid contents were determined following the methods described by Lichtenthaler (1987) [46]. Fresh leaf samples (~0.2 g) were extracted in 10 ml of 80% acetone and analyzed at wavelengths of 663, 645, and 470 nm using a T6 New Era Spectrophotometer (General Purpose, Beijing, China). Each experiment was conducted with three biological replicates.

### Transmission electron microscopy

The second true leaf (new leaf) and the second fully unfolded true leaf (old leaf) from both *gret1* and ‘T12404’ were collected at 30 and 60 DAG, respectively, for TEM analysis. Leaf sections were fixed in 2.5% glutaraldehyde at 4°C for 12 h, then dehydrated in a graded ethanol series and embedded in Spurr resin. Thin sections were prepared using a Leica EMUC6 ultramicrotome and examined with a Hitachi H-7700 transmission electron microscope (HITACHI, Tokyo, Japan) [47].

### Map-based cloning of *SlPPR138* gene

To map the *SlPPR138* locus, we conducted genetic analysis using an F_2_ mapping population derived from crossing between the *gret1* mutant and the WT ‘T12404’. This population consisted of 483 individuals, from which DNA samples were taken from 25 plants displaying a WT phenotype and 25 plants displaying a mutant phenotype. Four Illumina libraries, including two parental pools and two F_2_ pools, were individually constructed and sequenced using the Illumina HiSeq™ PE150 sequencer (Novogene Biotech Co., Ltd., Beijing, China). SNPs and insertions/deletions (InDels) were identified using the Genome Analysis Toolkit (GATK 3.8), with the Heinz 1706 genome version SL 4.0 as the reference (http://solgenomics.net). An SNP index was calculated as the ratio of reads with mutant SNPs to the total number of reads for each SNP, employing a sliding window approach. Δ(SNP-index) analysis was conducted as described by Chai *et al*. (2021) [48].

Homozygous mutation sites were screened, and the SNP index values for the two progeny pools were compared against one parent. The average index for each window was computed, and candidate intervals were identified. Only intervals meeting a 99% confidence threshold were selected. Candidate genes were then determined based on ANNOVAR annotation, focusing on significant mutation sites such as upstream variants, stop loss, stop gain, nonsynonymous mutations, and alternative splicing.

### Complementation and knockout of *SlPPR138*

For functional complementation of the *gret1* mutation, the full-length coding sequence (CDS) of *SlPPR138* (LOC Solyc03g025700) was amplified from WT young leaves. The amplified PCR product was then inserted into the pFGC1008–3 × HA vector to create the SlPPR138–1008 construct, where the SlPPR138 gene was controlled by the 35S CaMV promoter. This construct was introduced into *Agrobacterium tumefaciens* strain GV3101 and used for transforming *gret1* mutant plants through *Agrobacterium*-mediated transformation. Successful transgenic plants were confirmed by PCR amplification.

To further elucidate the role of SlPPR138, knockout plants were generated using CRISPR/Cas9 technology [49]. The process involved designing and synthesizing single-guide RNA (sgRNA) targeting SlPPR138, following guidelines provided at http://cbi.hzau.edu.cn/cgi-bin/CRISPR. This sgRNA was then inserted into the psg-Cas9-At-pMD18-T vector. The CRISPR vectors were delivered into the *A. tumefaciens* strain GV3101 and subsequently transferred into tomato plants of the ‘Ailsa Craig’ variety using the leaf-disc method [50].

### RNA isolation and quantitative real-time PCR

Total RNA was extracted from a variety of plant tissues, both mutant and WT, using the Plant RNA Kit (OMEGA, Norcross, GA, USA). This RNA was then converted into complementary DNA (cDNA) using the PrimeScript™ RT Reagent Kit (Takara, Kusatsu, Japan). qRT-PCR was conducted using SYBR^®^ Green Realtime PCR Master Mix (Toyobo, Osaka, Japan) on the Bio-Rad CFX96 Real-Time PCR System (Bio-Rad, Hercules, CA, USA). The expression levels of the target genes were standardized against the internal reference gene *SlUbi3* and calculated using the 2^-ΔΔCt^ method [51]. Each qRT-PCR analysis included three biological and three technical replicates to ensure accurate results.

### Subcellular localization analyses

The *SlPPR138* coding regions, excluding the stop codon, were amplified using genomic DNA as the template and cloned into the pFGC-eGFP vector [52]. Tomato protoplasts were prepared as previously described [52]. After 16 h of dark culture at 28°C, the green fluorescent protein (GFP) and chlorophyll fluorescence signals of the chloroplast, along with the NLS-red fluorescent protein (RFP), were observed and photographed using a Nikon confocal laser scanning microscope (A1-SHS, Nikon, Tokyo, Japan).

### Chloroplast RNA editing analysis

Extracted total RNA from young leaves of mutant and WT plants, reverse transcribed using M-MLV reverse transcriptase (Invitrogen). RNA editing site analysis was conducted as described by Kahlau *et al*. [30]. Specific fragments containing editing sites were amplified using reverse transcription RT-PCR and then sequenced.

All primers used in this study are listed in Table S1.

## Supplementary Material

Web_Material_uhaf193

## Data Availability

The raw sequencing data from the BSA-seq have been uploaded to NCBI under accession number PRJNA1272591.
