# Supplementary material for: SlPPR138-mediated RNA editing of rpoC1 is essential for chloroplast development in tomato
Source: Hortic Res. 2025 Jul 28;12(10):uhaf194. doi: 10.1093/hr/uhaf194 (PMC12541717; doi:10.1093/hr/uhaf194)
Supplement: Web_Material_uhaf193 [file web_material_uhaf193.zip › Description of figureS and TableS.docx]

**Table S1.** All primers used in this study.

|  | Number of normal leaf color plants | Number of yellow leaf color plants | segregation ratio | Total plant number |
| --- | --- | --- | --- | --- |
| Actual number of plants | 378 | 105 | 3.6:1 | 483 |
| Theoretical number of plants | 362.25 | 120.75 | 3:1 | 483 |
| X^2^ | 2.57 |  |  |  |

**Table S2.** The mutant was controlled by single recessive gene.

|  | Variant | Chrom | Pos | Ref | Alt | SNPindex_405-G | SNPindex_405-Y | Delta_SNPindex(405-Y-405-G) |
| --- | --- | --- | --- | --- | --- | --- | --- | --- |
| Solyc03g025700.2 | nonsynonymous | SL4.0ch03 | 3101674 | T | C | 0 | 0.58 | 0.58 |
|  | intergenic region | SL4.0ch03 | 21436290 | T | A | 0.125 | 0.6364 | 0.5114 |
|  | intergenic region | SL4.0ch03 | 34918517 | T | A | 0.0952 | 0.6417 | 0.5518 |
|  | intergenic region | SL4.0ch03 | 35711728 | C | G | 0 | 0.565 | 0.565 |

**Table S3.** There were four SNPs between the two pools between SL4.0ch03_1 and SL4.0ch03_65298490

**
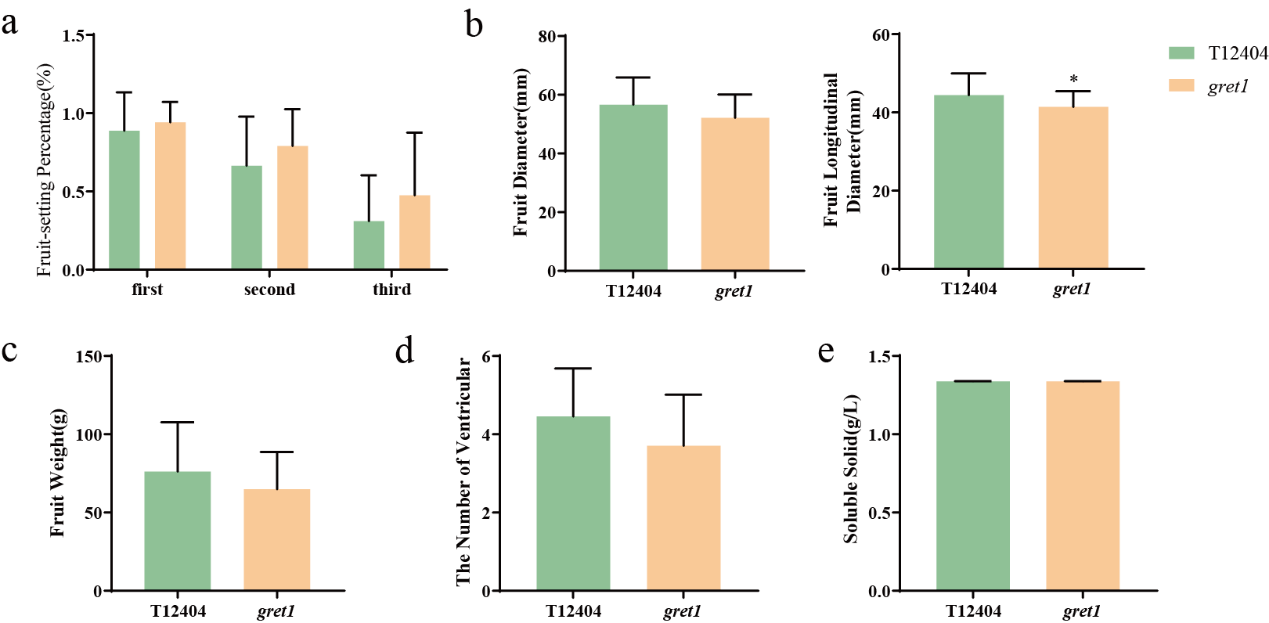
**

**Fig S1. The fruit traits in wild-type (T12404) and *gret1* mutant** **in tomato plants**

a, The percentage of fruit set in the first, second and third internode respectively. b, Fruit transverse and longitudinal diameter of fruit. c, Fruit weight. d, Locule number. e, Fruit soluble solids content.

**
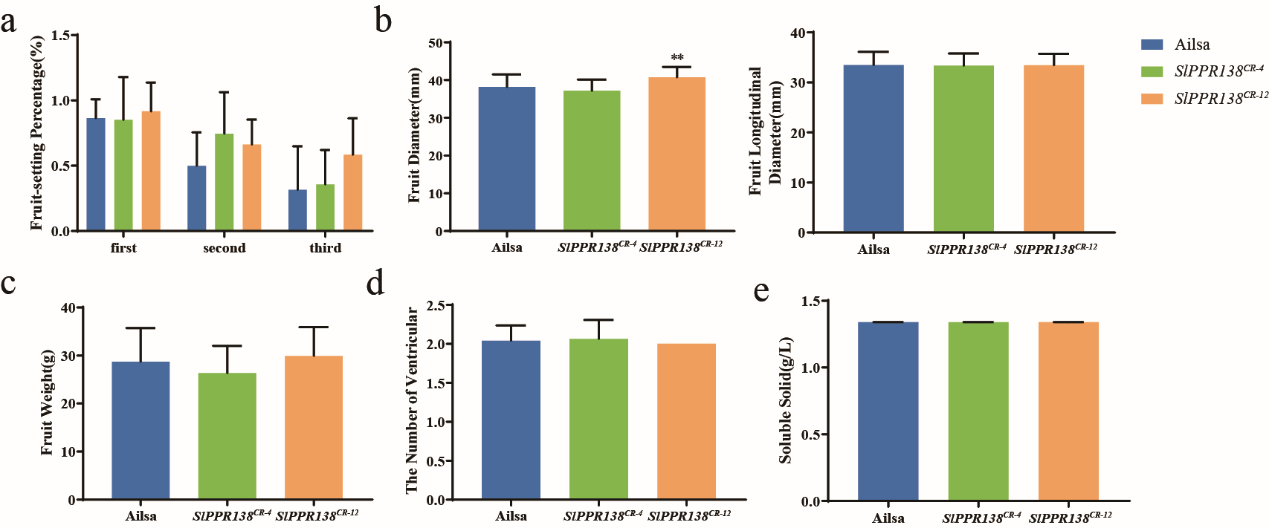
**

**Figure S2. Comapration of the fruit traits between SlOTP70 knockout lines and wild-type (AC) plants.**

a, The percentage of fruit set in the first, second and third internoderespectively. b, Fruit transverse and longitudinal diameter of fruit. c, Fruit weight. d, Locule number. e, Fruit soluble solids content.

**
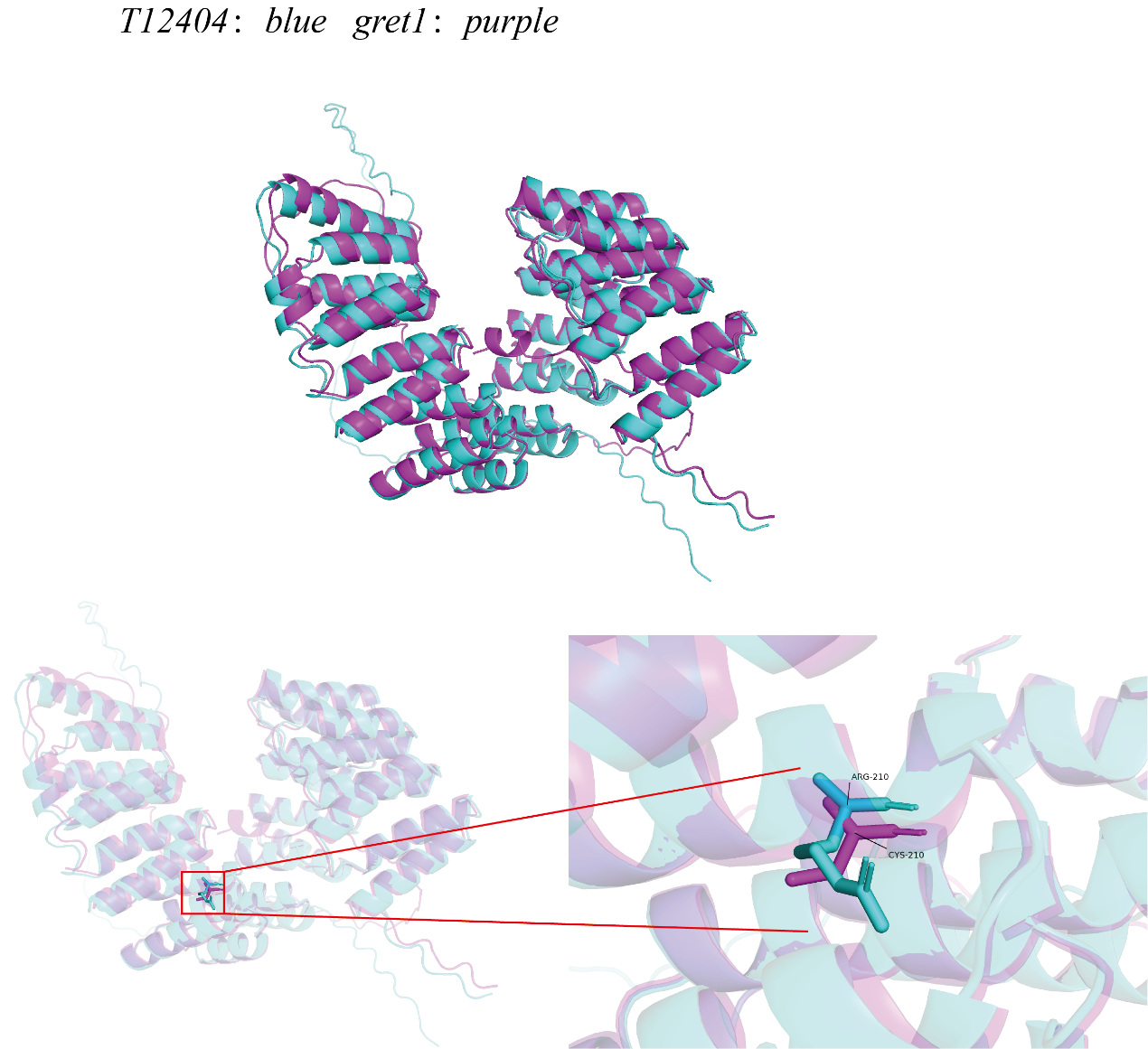
**

**Figure S****3.** Protein structure prediction of *gret1* and WT by AlphaFold3. The highlighted part is the location of the mutant amino acid.

**
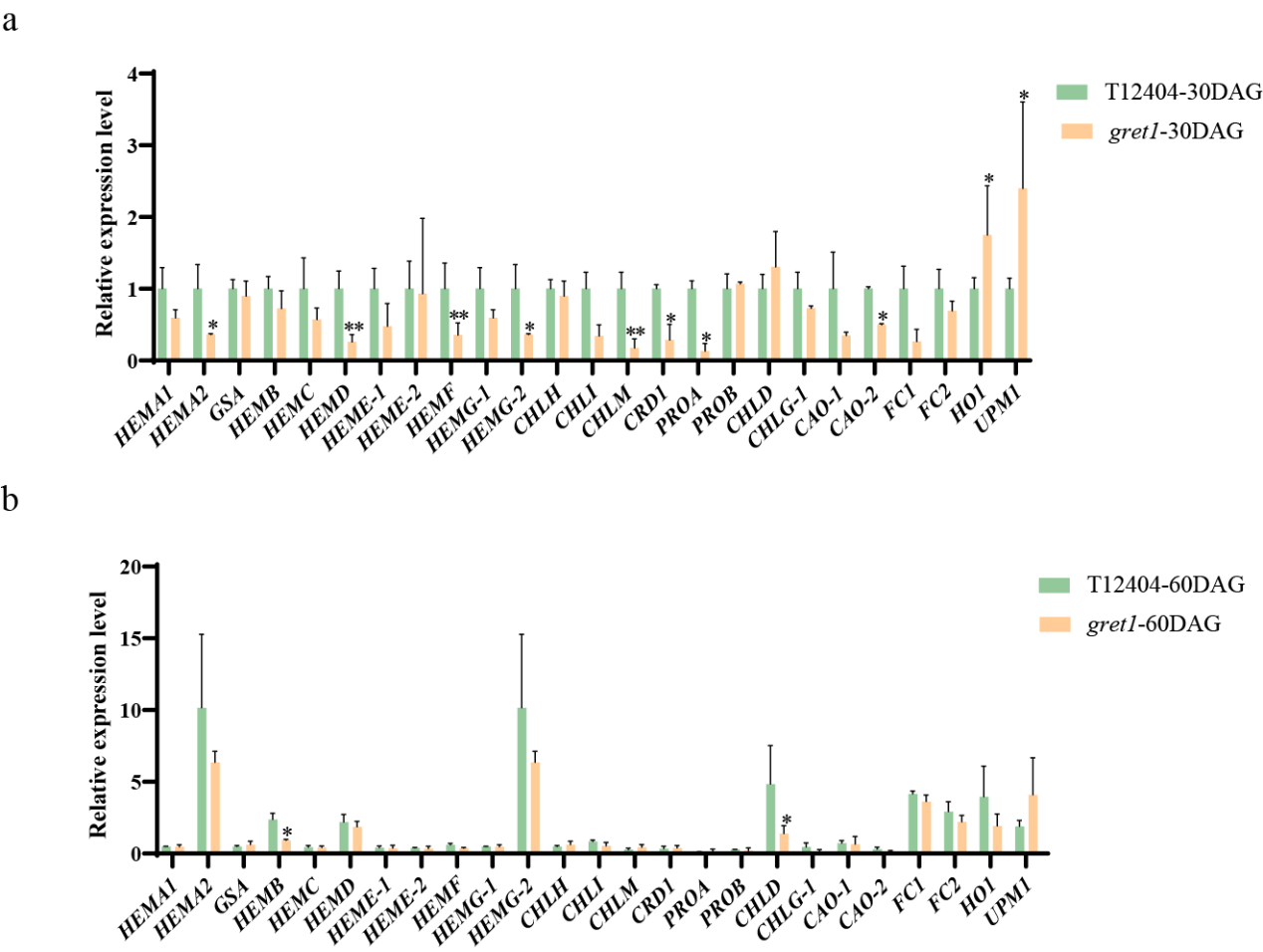
**

**Fig S4.** **Expression of** **chlorophyll biosynthesis-related genes in wild-type (T12404) and gret1 mutant leaves.**

a, qRT-PCR analysis of chlorophyll biosynthesis-related gene expression in wild-type (T12404) and gret1 leaves at 30 DAG (approximately the fourth-leaf stage).

b, qRT-PCR analysis of chlorophyll biosynthesis-related gene expression in wild-type (T12404) and gret1 leaves at 60 DAG (approximately the 11th-leaf stage).

Data are presented as the mean ± SD of 5 biological replicates. Asterisks above the bars indicate statistically significant differences (Student’s t-test: * P < 0.05, **P < 0.01).

**
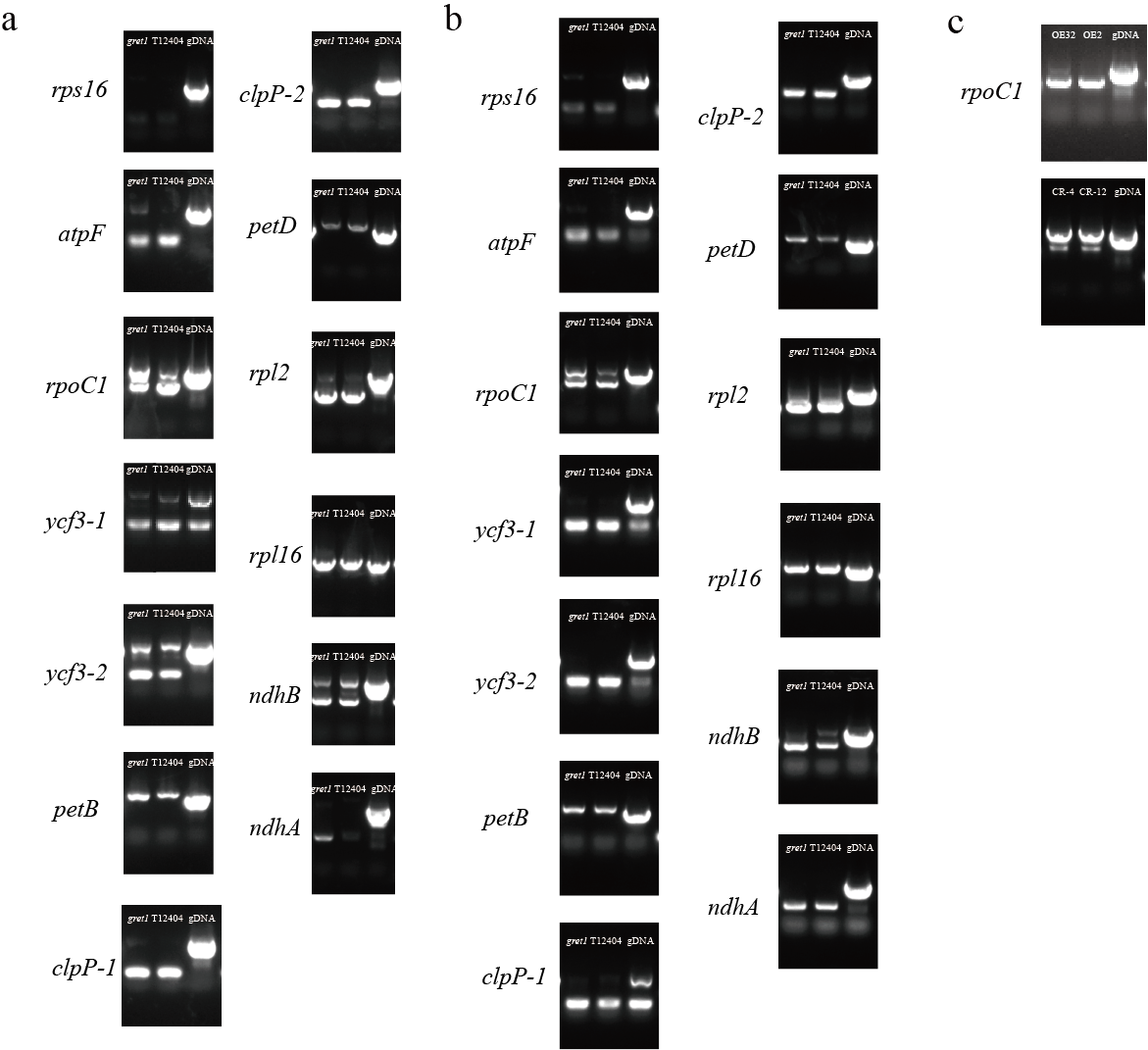
**

**Fig S5.** **Analysis of the chloroplast RNA splicing in wild-type (T12404) and *gret1* mutant leaves.**

a, RT-PCR analysis of the intron splicing of chloroplast transcript of young leaves in wild-type (T12404) and *gret1* mutant at 30 DAG;

b, RT-PCR analysis of the intron splicing of chloroplast transcript of mature leaves in wild-type (T12404) and *gret1* mutant at 30 DAG;

c, RT-PCR analysis of the intron splicing of chloroplast transcript of young leaves in SlPPR138 knockout and overexpressing plants at 30 DAG.

All PCR products were confirmed by sequencing.


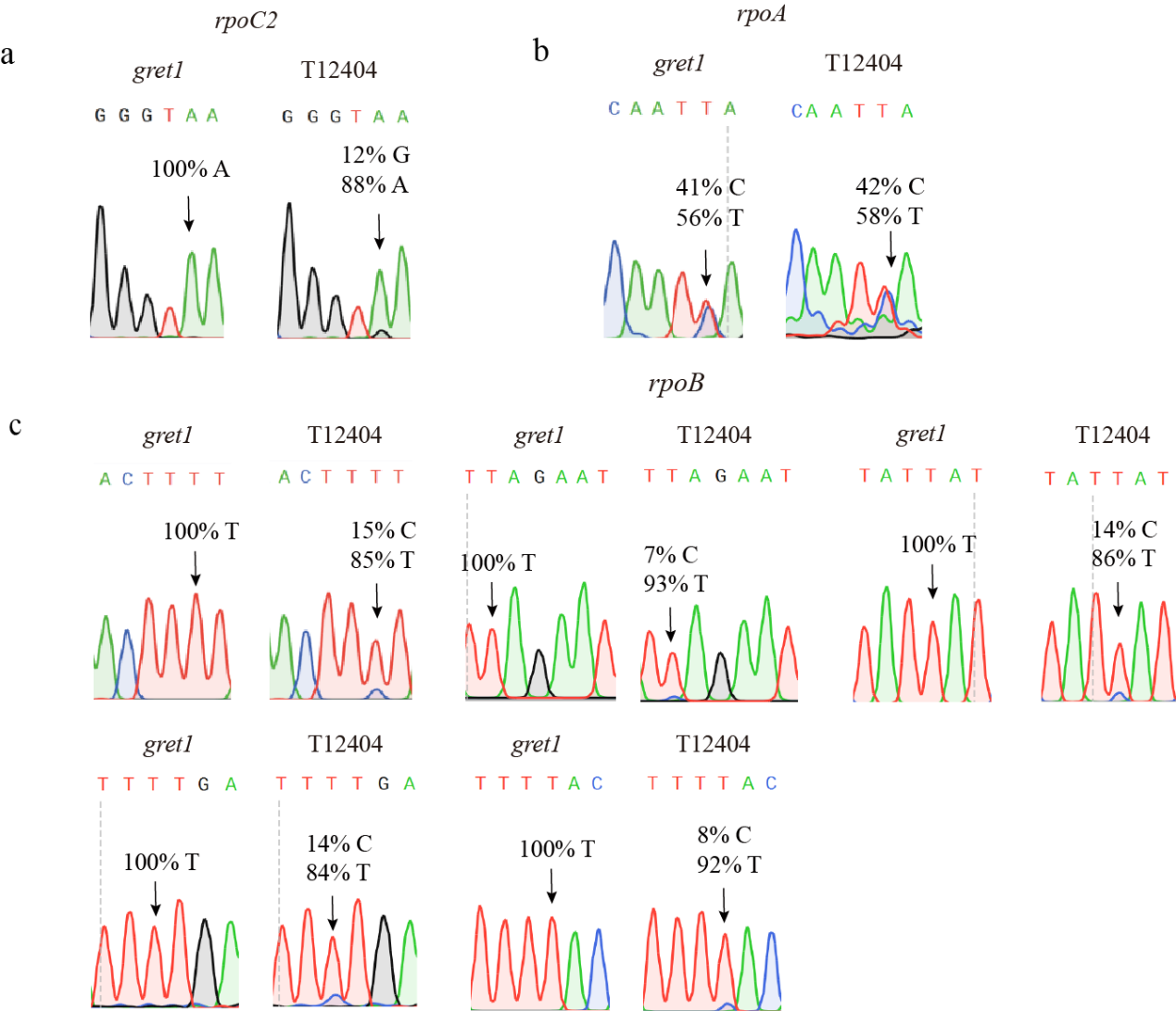


**Fig S6. RNA Editing Efficiency of rpoC2, rpoA and rpoB in wild-type (T12404) and *gret1* mutant leaves.**

a, Sequence chromatograms of plastid RNA editing sites of rpoC2 in young leaves of wild-type (T12404) and *gret1* plants at 30 DAG.

b, Sequence chromatograms of plastid RNA editing sites of rpoA in young leaves of wild-type (T12404) and *gret1* plants at 30 DAG.

c, Five sites of sequence chromatograms of plastid RNA editing sites of rpoB in young leaves of wild-type (T12404) and *gret1* plants at 30 DAG.


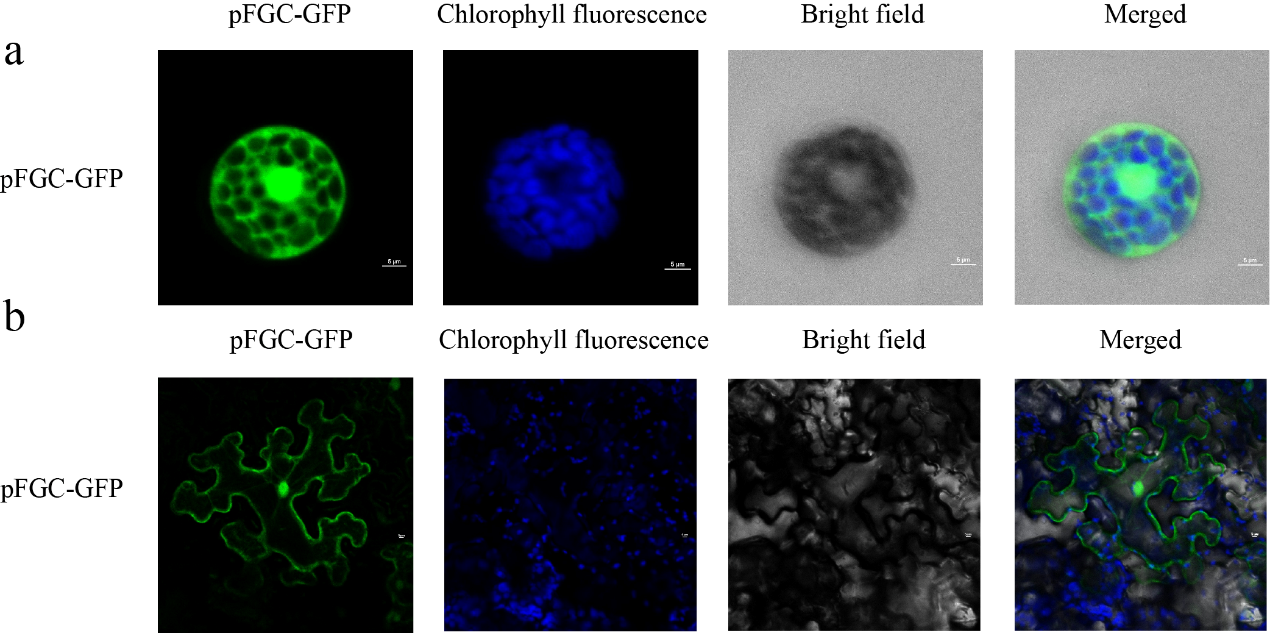


**Fig S7. Subcellular localization analysis of the GFP empty vector (pFGC-GFP).**

a, Subcellular location of the pFGC-GFP fusion protein in leaf epidermal cells of tomato protoplasts. GFP, green fluorescent protein; Chlorophyll, chlorophyll autofluorescence. Merge, merge images of GFP and chlorophyll autofluorescence. Scale Bars: 5 µm. b, Subcellular location of the pFGC -GFP fusion protein in the leaf epidermal cells of *N. benthamiana*. Scale Bars: 5 µm.
