# Supplementary figures and images for: SlPPR138-mediated RNA editing of rpoC1 is essential for chloroplast development in tomato
Source: Hortic Res. 2025 Jul 28;12(10):uhaf194. doi: 10.1093/hr/uhaf194 (PMC12541717; doi:10.1093/hr/uhaf194)

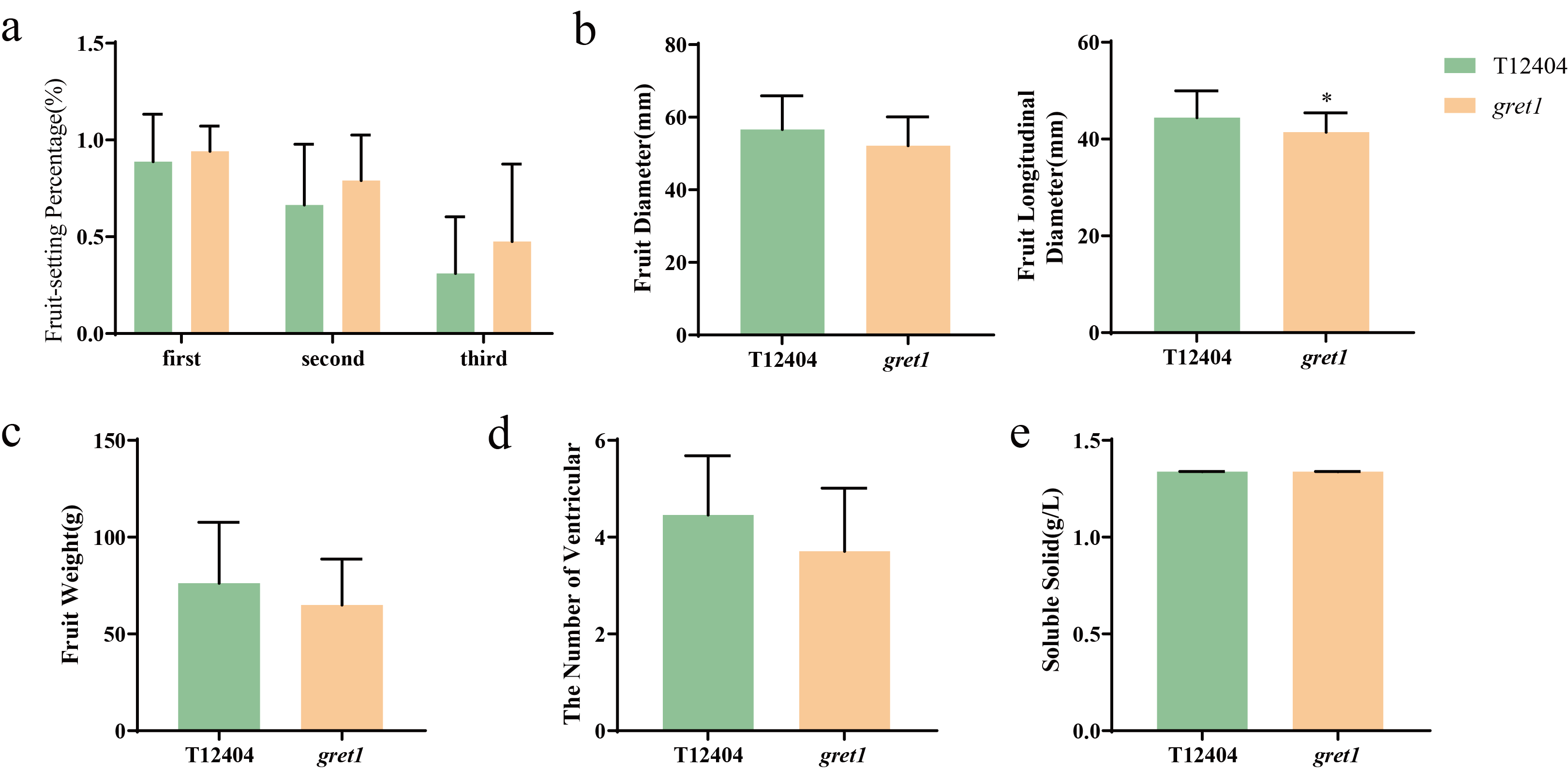

Supplement: Web_Material_uhaf193 [file web_material_uhaf193.zip › FigS1.tif]

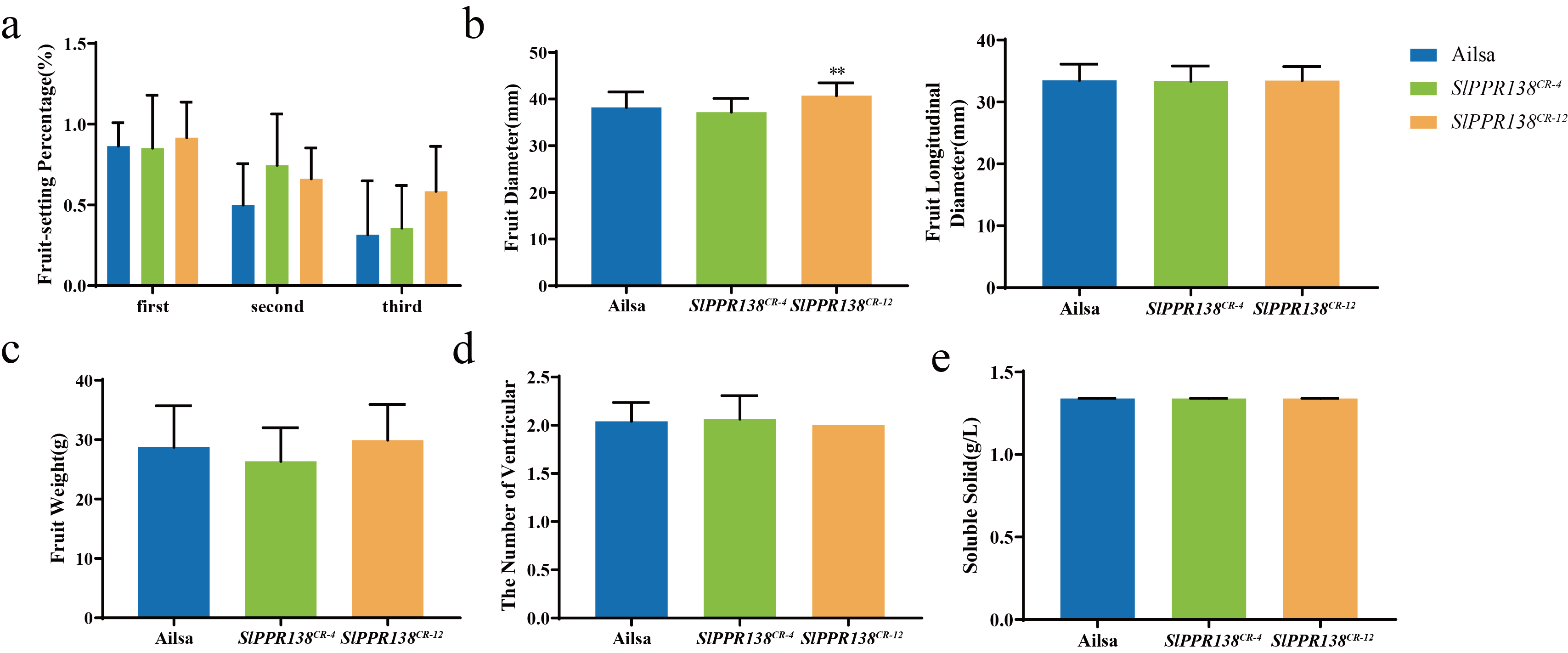

Supplement: Web_Material_uhaf193 [file web_material_uhaf193.zip › FigS2.tif]

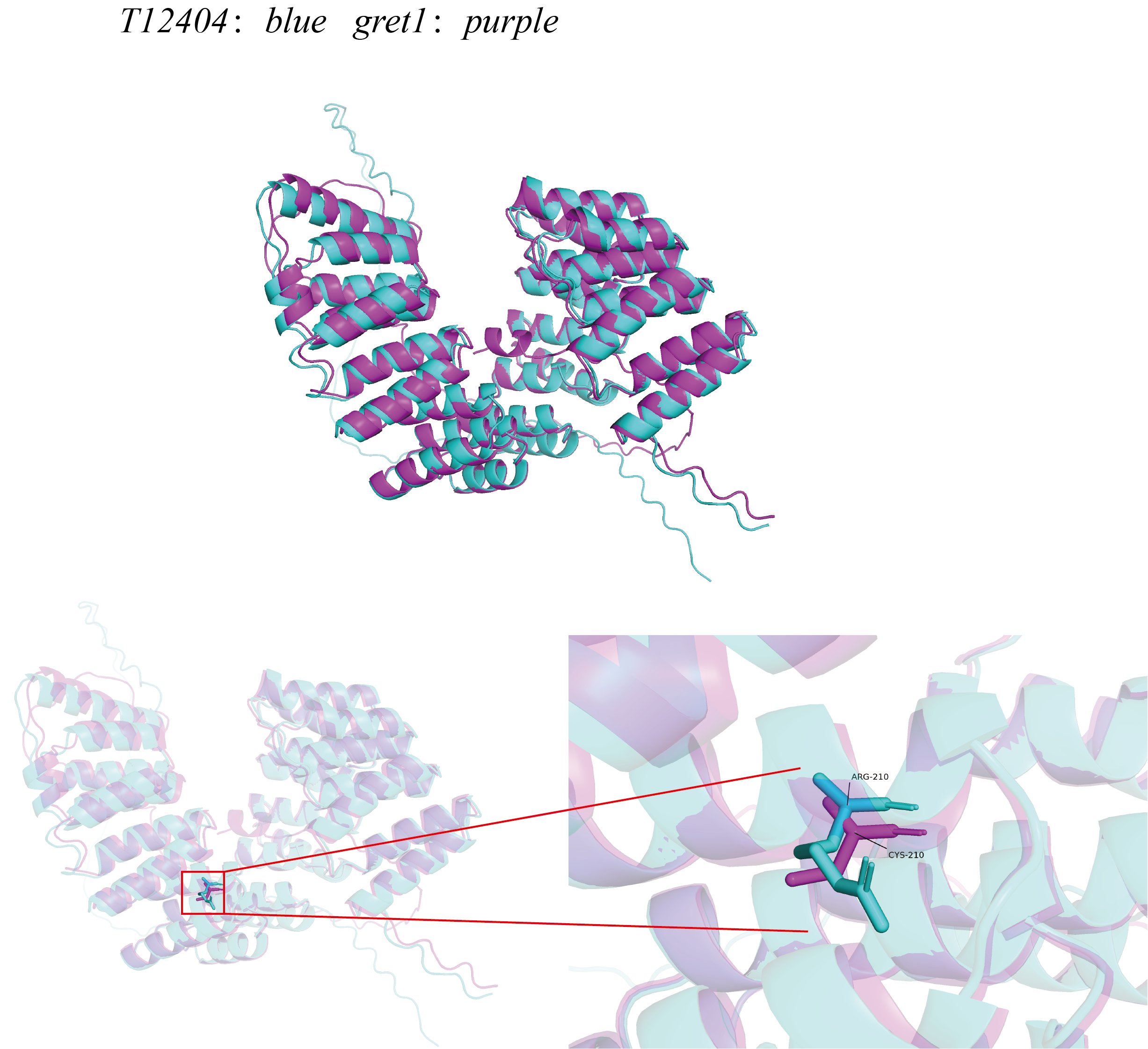

Supplement: Web_Material_uhaf193 [file web_material_uhaf193.zip › FigS3.tif]

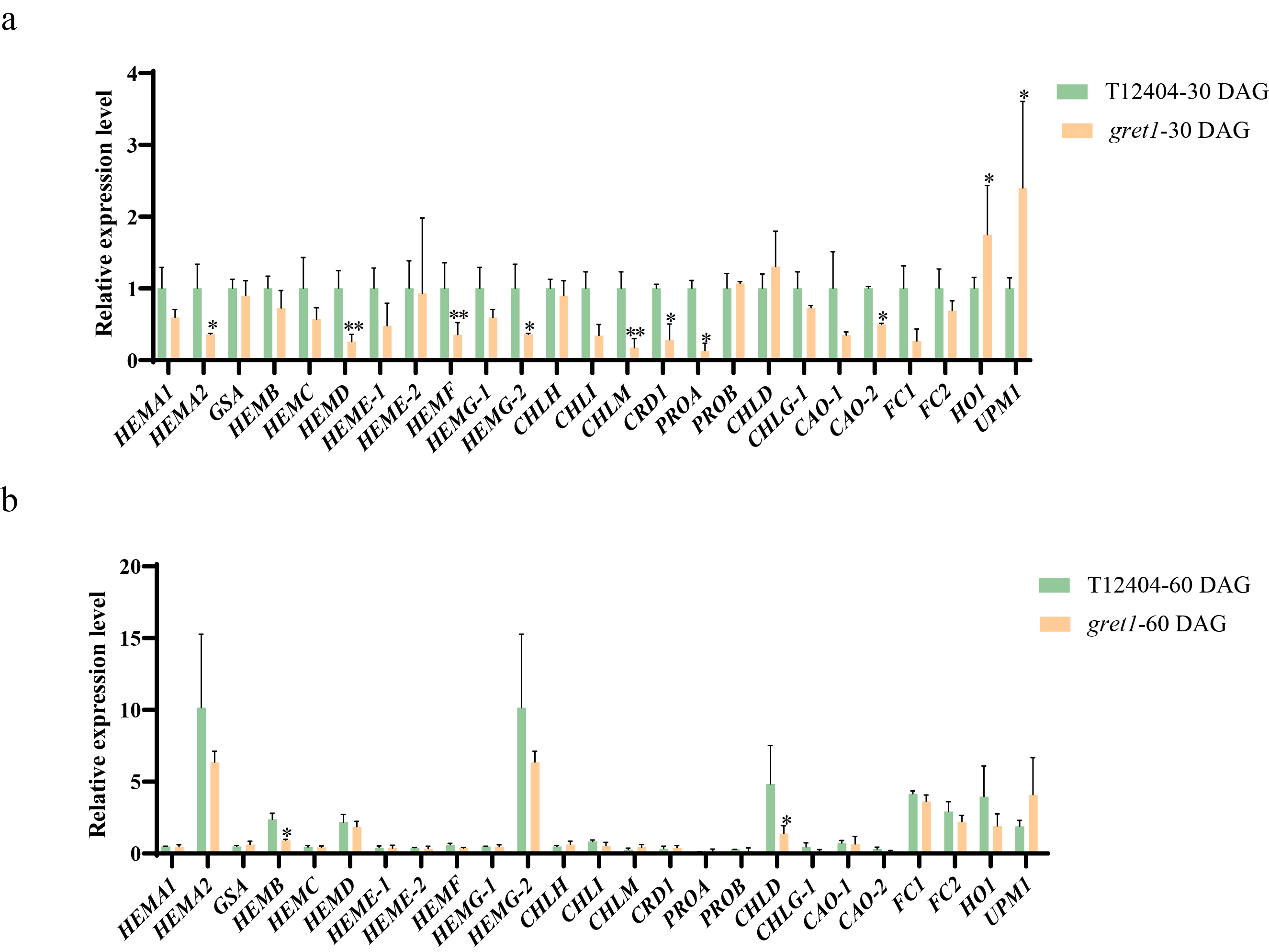

Supplement: Web_Material_uhaf193 [file web_material_uhaf193.zip › FigS4.tif]

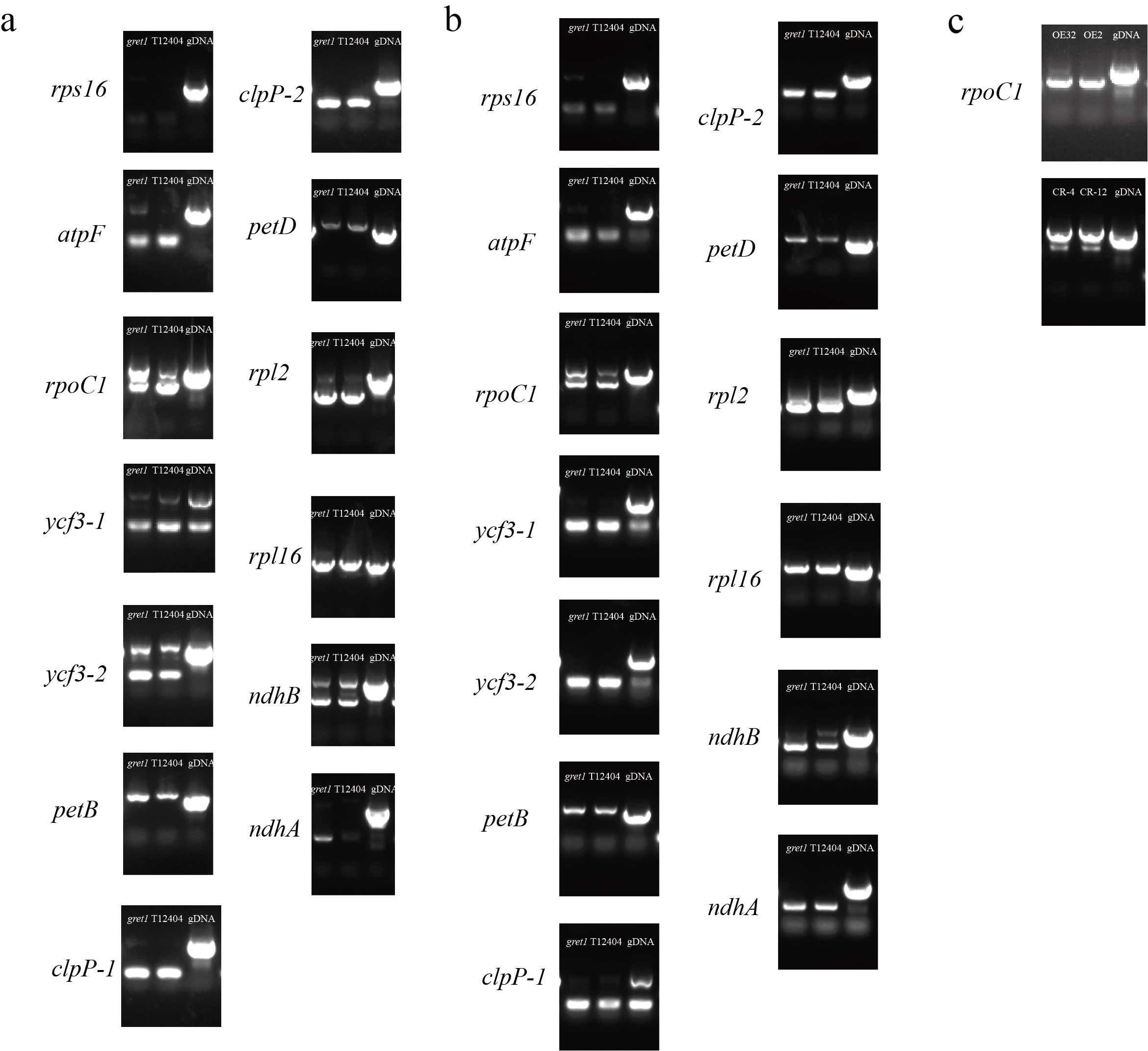

Supplement: Web_Material_uhaf193 [file web_material_uhaf193.zip › FigS5.tif]

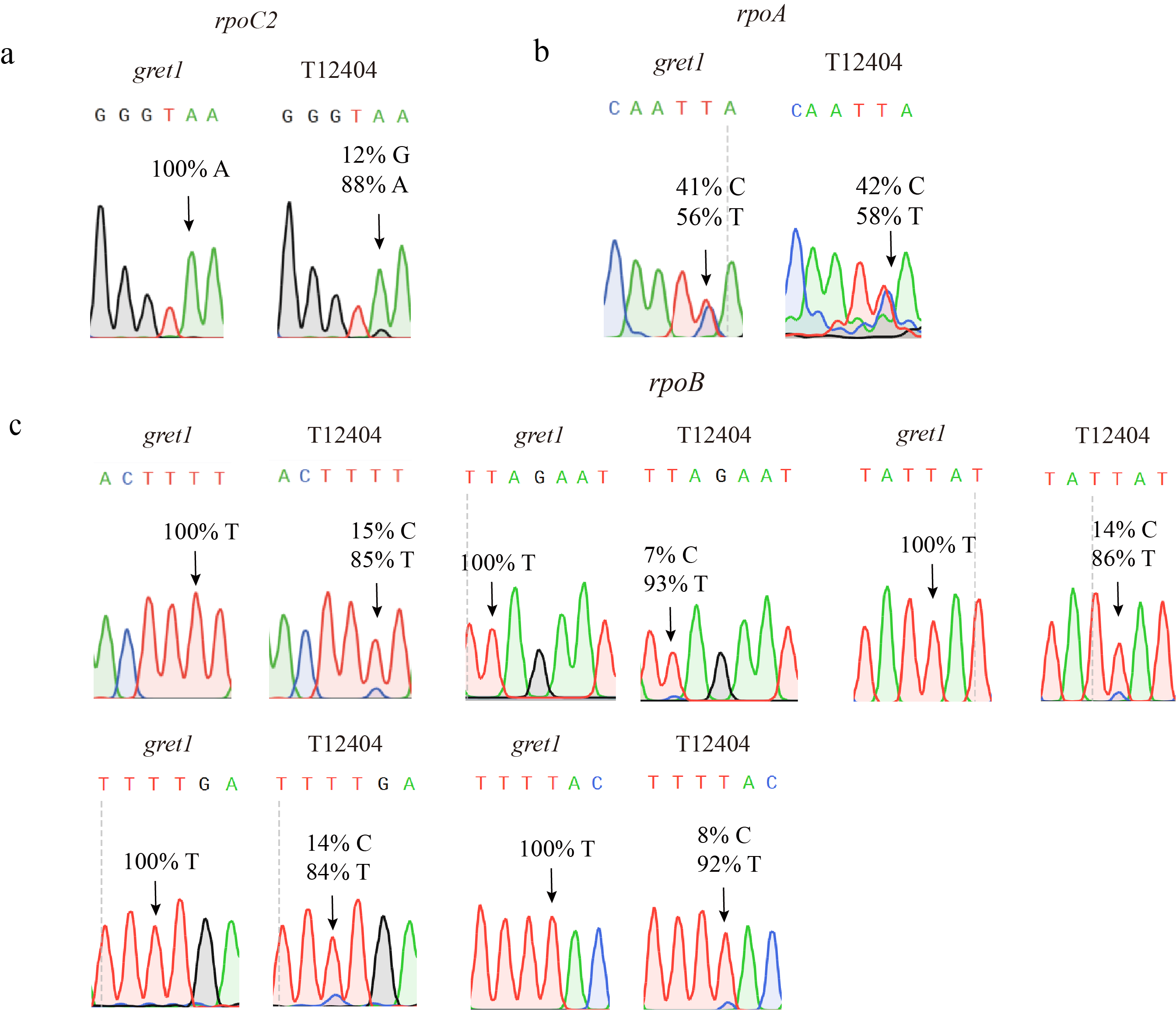

Supplement: Web_Material_uhaf193 [file web_material_uhaf193.zip › FigS6.tif]

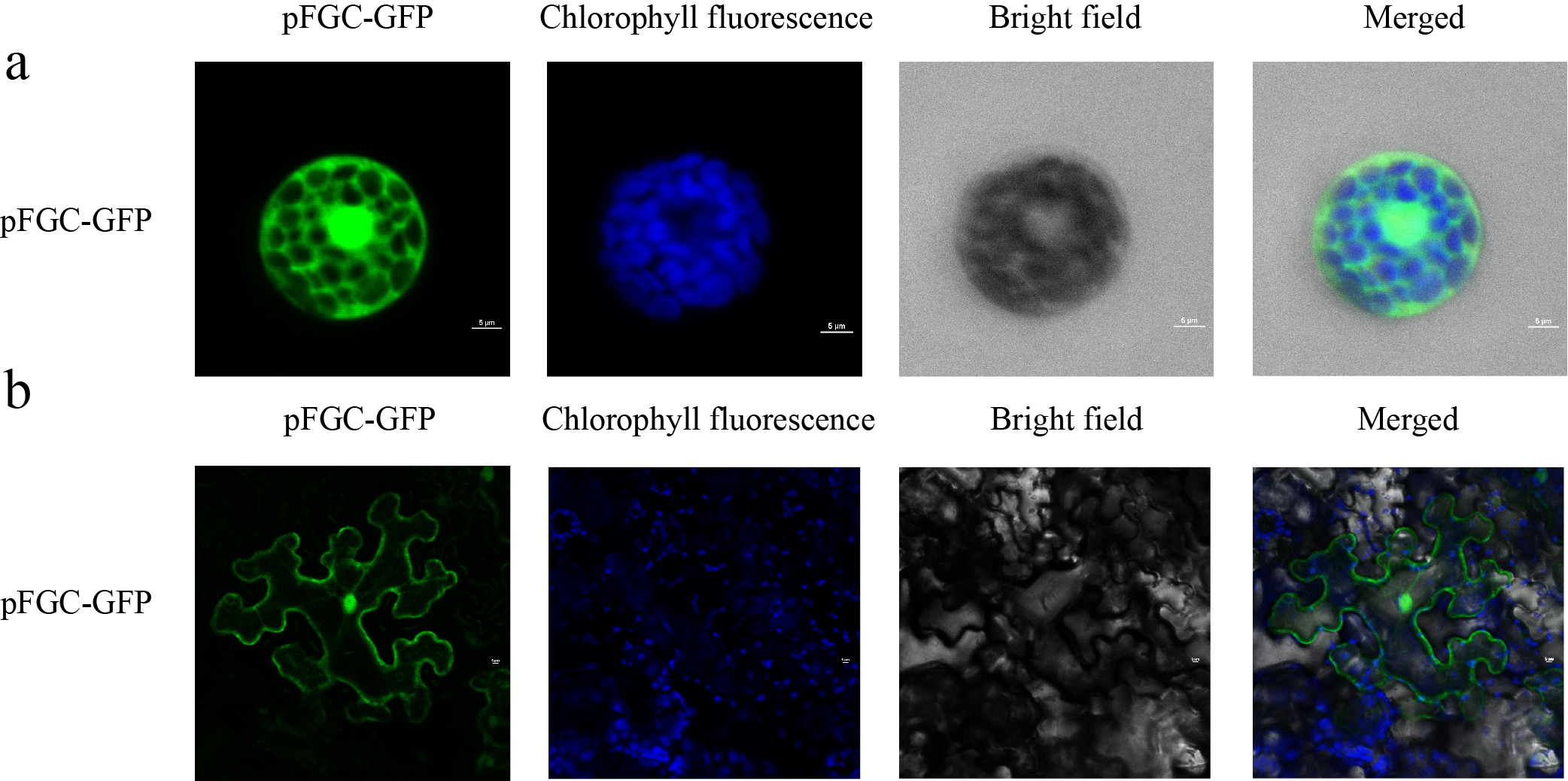

Supplement: Web_Material_uhaf193 [file web_material_uhaf193.zip › FigS7.tif]
